# The Use of Artificial Intelligence for Detecting and Predicting Atrial Arrhythmias Post Catheter Ablation

**DOI:** 10.31083/j.rcm2408215

**Published:** 2023-07-31

**Authors:** Poojesh Nikhil Lallah, Chen Laite, Abdul Basit Bangash, Outesh Chooah, Chenyang Jiang

**Affiliations:** ^1^Department of Cardiology, Sir Run Run Shaw Hospital, School of Medicine, Zhejiang University, 310016 Hangzhou, Zhejiang, China; ^2^Department of Radiology, Sir Run Run Shaw Hospital, School of Medicine, Zhejiang University, 310016 Hangzhou, Zhejiang, China

**Keywords:** artificial intelligence, atrial fibrillation, atrial flutter, atrial tachycardia, atrial arrhythmias, post ablation, catheter ablation, machine learning, deep learning

## Abstract

Catheter ablation (CA) is considered as one of the most effective methods technique for eradicating persistent and abnormal cardiac arrhythmias. Nevertheless, in some cases, these arrhythmias are not treated properly, resulting in their recurrences. If left untreated, they may result in complications such as strokes, heart failure, or death. Until recently, the primary techniques for diagnosing recurrent arrhythmias following CA were the findings predisposing to the changes caused by the arrhythmias on cardiac imaging and electrocardiograms during follow-up visits, or if patients reported having palpitations or chest discomfort after the ablation. However, these follow-ups may be time-consuming and costly, and they may not always determine the root cause of the recurrences. With the introduction of artificial intelligence (AI), these follow-up visits can be effectively shortened, and improved methods for predicting the likelihood of recurring arrhythmias after their ablation procedures can be developed. AI can be divided into two categories: machine learning (ML) and deep learning (DL), the latter of which is a subset of ML. ML and DL models have been used in several studies to demonstrate their ability to predict and identify cardiac arrhythmias using clinical variables, electrophysiological characteristics, and trends extracted from imaging data. AI has proven to be a valuable aid for cardiologists due to its ability to compute massive amounts of data and detect subtle changes in electric signals and cardiac images, which may potentially increase the risk of recurrent arrhythmias after CA. Despite the fact that these studies involving AI have generated promising outcomes comparable to or superior to human intervention, they have primarily focused on atrial fibrillation while atrial flutter (AFL) and atrial tachycardia (AT) were the subjects of relatively few AI studies. Therefore, the aim of this review is to investigate the interaction of AI algorithms, electrophysiological characteristics, imaging data, risk score calculators, and clinical variables in predicting cardiac arrhythmias following an ablation procedure. This review will also discuss the implementation of these algorithms to enable the detection and prediction of AFL and AT recurrences following CA.

## 1. Introduction

Atrial arrhythmias are abnormal heart rhythms that occur in the upper right and left cardiac chambers. Typically, a normal sinus rhythm begins with an impulse generated at an optimal discharge rate in the sinoatrial node. The impulse then travels through the atrioventricular node, the bundle of His and to the left and right bundle branches before reaching the Purkinje fibres. Any generated impulse that discharges either too quickly, too slowly or out of order contributes to the emergence of an arrhythmia [1]. Arrhythmias can be categorised as either slow or fast heart rhythms, the significance of which can be accentuated by the presence of a structural heart disease. This can lead to severe complications such as worsening arrhythmias, stroke, heart failure, or even death [2]. Therefore, treatment of cardiac arrhythmias is of paramount importance. Typically, pharmacological methods or catheter ablation (CA) are used. Evidence suggests that CA employing heat (radio frequency) or cold (cryoablation) techniques to create scars in the heart to block abnormal impulses is superior to standard pharmaceutical treatments [3, 4]. Ablation is successful when no arrhythmias causing symptoms persist for more than 30 seconds following the procedure [5]. However, in some cases, these arrhythmias may persist or worsen after CA, necessitating another ablation procedure.

In a post-ablation setting, physicians deal with three recurrent arrhythmias: atrial fibrillation (AF), atrial flutter (AFL), and atrial tachycardia (AT) [6, 7, 8]. These recurring arrhythmias are often detected after the ablation procedure, either through follow-up visits or patient complaints of chest pain or palpitations. To detect any significant changes that may predispose to recurring arrhythmias, patients must undergo a multitude of tests and examinations, including electrocardiogram (ECG), cardiac imaging tests such as transesophageal echocardiography (TEE), transthoracic echocardiography (TTE) and cardiac magnetic resonance (CMR), laboratory testing such as NT-pro B-type Natriuretic Peptide (NT-proBNP), and calculated risk score evaluations. This, in turn, leads to a significant number of follow-up visits. These visits can be time consuming and costly, particularly if the causes of the arrhythmias are obscure, challenging to detect, or unidentified.

In order to deal with these recurrences, it is vital to predict potential recurrences following any ablation treatment. Arrhythmia patterns on ECG, specifically p-wave morphology, have been demonstrated to be predictive of future arrhythmia recurrence [9, 10]. Changes observed on cardiac imaging modalities such as atrial enlargement and impaired function have also been linked to the development of these arrhythmias [11, 12, 13]. Risk calculators were developed and implemented to assess the likelihood and prediction of these recurring atrial arrhythmias using a combination of data acquired from cardiac imaging and electrophysiological studies, as well as easily acquired variables such as age, gender, smoking status, hypertension and other comorbidities [14, 15, 16, 17]. Computational or manual interventions have been used to establish a relationship between these features using a flowchart approach and the likelihood of recurrence using statistical testing [18]. However, there are an array of drawbacks to physically examining these traits in order to identify or predict any potential risks of arrhythmia recurrences, including (i) omitting some important parameters, (ii) not using enough data to accurately produce a conclusive result, and (iii) failing to identify ECG changes or changes on imaging modalities that predispose to the development of an arrhythmia. This ultimately prompted the search for a better substitute for these computational or observational methods, which led to the development of AI.

Utilisation of artificial intelligence in clinical investigations is no longer uncommon. It has been used to analyse massive data sets consisting of health records, medical imaging, population data, and clinical trial data to uncover correlating patterns, predict outcomes, and provide better patient management strategies [19]. Implementing AI for screening cardiac disease is a subject of intense debate in the medical and clinical research communities. The standard methods for the screening for atrial arrhythmias are costly, time-consuming, and a financial strain to patients. Consequently, machine learning (ML) and deep learning (DL) (the latter also known as artificial neural network [ANN]), two major subfields of AI, can be utilized for this purpose because they are non-invasive, faster and more effective than conventional methods. This makes AI an appealing option to both the patient and the physician.

As more data in hospitals is gradually being digitalized, the role of AI is growing. AI can access hospital and web databases, learn from them, and employ appropriate algorithms to calculate outcomes from any abnormalities that they have previously analysed or detected in a real-world clinical setting. ML and ANN have been shown to effectively and simultaneously analyse electrocardiograms, imaging findings, and variables from risk score calculators in detecting and predicting atrial arrhythmias, producing results comparable to those of a human expert [20, 21, 22, 23]. Their post-ablation use for predicting recurring arrhythmias is under intensive investigation due to their ability to detect subtle changes that a typical physician may overlook or find incomprehensible [24, 25, 26]. This review examines and discusses AI technology that has been used in prior studies for the detection and prediction of atrial arrhythmias following CA, based on parameters from electrophysiology, medical imaging, and variables from risk score calculators to target specific post-ablation recurrences of AF as well as AFL and AT, both of which have been previously overlooked.

## 2. Types of AI Algorithms Involved in Medical Studies

AI uses advanced computerised methods to perform tasks capable of rivalling human intelligence. They can be used for visual interpretation, speech recognition, decision-making, and translation of languages. Medical tasks are associated with clinical decisions and imaging analysis to search for medical data that benefit healthcare outcomes. AI methods principally consist of ML and ANN.

**Fig. 1.**
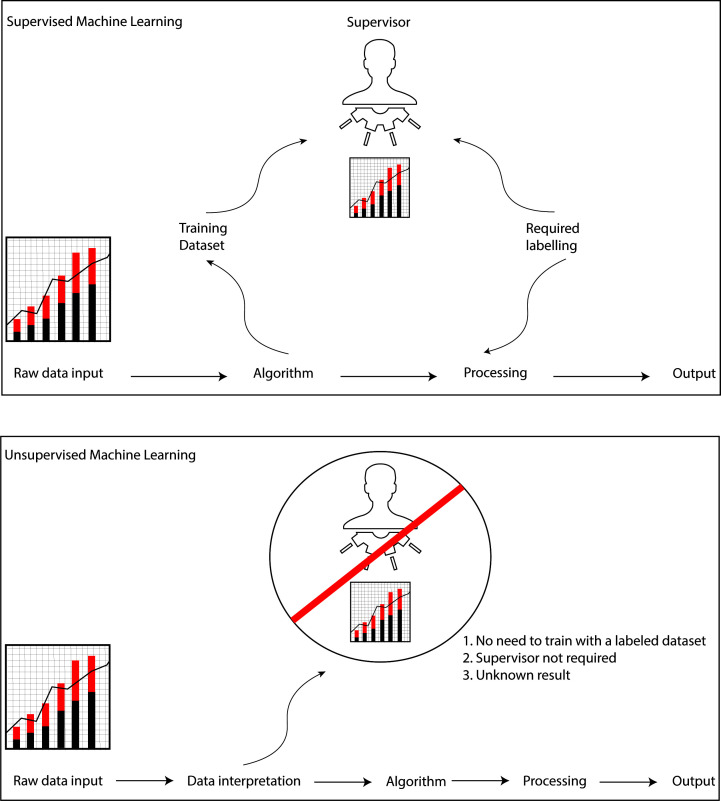
**The basic differences between a supervised ML and an unsupervised ML**. ML, machine learning.

ML is based on algorithms that develop automatically through a gradual process of learning from data, visualising patterns, and making judgments [19]. It parses and learns from input data using a combination of computational and statistical methods to produce an output that is not visible by conventional statistical techniques. There are two types of ML in medical science: supervised ML and unsupervised ML. A general overview on the differences between the two variants of ML has been illustrated in Fig. 1. Supervised ML is distinguished by how it trains computers to classify data accurately or predict outcomes using labelled (arranged) datasets. The algorithms involved in this ML deal mostly with classification or regression purposes. Standard algorithms include Support Vector Machine (SVM), Logistic regression, Least Absolute Shrinkage and Selection Operator (LASSO) regression, Naive Bayes, Random Forest, and k-nearest neighbour (k-NN). In medicine, supervised ML has been widely used, particularly to determine which features can help doctors make correct diagnoses of diseases they suspect. A study was conducted with a supervised ML algorithm trained with several ECG features to distinguish AFL from other atrial arrhythmias, and the ML algorithm performed very well in this task [27]. Unsupervised ML uses unlabelled input data to infer patterns by extracting features from raw data without the need to rearrange the data. The most common purpose of unsupervised ML consists of clustering or association issues. Its ability to process results from data that has not been labelled makes it more and more important in medicine, primarily image analysis. K-means clustering is the most employed method of unsupervised ML in clinical studies [28, 29].

ANN or DL, is a subset of ML, inspired mainly by the human nervous system. These types of AI models consist of three primary layers: an input layer, a hidden layer, and an output layer, with each layer consisting of several nodes. The input layer deals with data input, such as variables, signals, or images, while the hidden layer is involved in altering weights during training based on accurate or incorrect judgments as it processes the input data. The output layer will display the processed data, which consists of the best-estimated value or probability [19, 30]. A slight difference between DL and ANN is that DL has more hidden layers than ANN. However, both entities may exist as feedforward neural networks and backpropagation. A feedforward neural network occurs when data is fed to all the nodes in the next layer rather than circulating within the layer. In backpropagation, data circulates within the same layer or is sent back to the previous layer [31]. DL can be also split into supervised and unsupervised DL. Supervised DL uses labelled data, such as clinical variables, images, or signals, to perform data processing and calculations while unsupervised DL involves extracting features from an unlabelled dataset without human intervention.

Convolutional Neural networks (CNN) and Recurrent Neural Networks (RNN) are the common forms of DL models used in medical science. CNN is a supervised DL model, which has been recognized for image and signals analysis, such as ECG signal analysis and cardiac imaging [32, 33, 34, 35]. RNN is most suited for data sequencing and is primarily used in time series analysis, handwriting recognition, and machine translation, making it useful for studies involved in prediction or prognosis [36]. This type of DL algorithm can exist as supervised and unsupervised. Autoencoders are unsupervised DL algorithms primarily associated with medical studies [37]. The key differences of DL models over ML models include their ability to learn high-level features from data and eliminate pre-processing of data involved with ML models. However, these models require large datasets to produce acceptable results.

The field of ML and DL techniques is vast. For a better understanding of these methods, the various supervised and unsupervised ML and DL algorithms that are currently being employed in studies, using AI in medicine, have been compiled. Table 1 (Ref. [19, 38, 39, 40, 41, 42, 43, 44, 45, 46, 47, 48, 49]) provides a brief summary of the names of commonly used AI algorithms used in medical studies, along with their advantages and disadvantages. This may aid in understanding why some AI algorithms are better suited for particular types of investigations.

**Table 1.** **A summary of the benefits and limitations about the types of supervised and unsupervised machine and deep learning algorithms mostly employed in medical studies**.

Types of AI	AI algorithms	Benefits	Limitations	References
Supervised ML	SVM	Strong generalisation ability with a good discriminative power	Low competence when dealing with large samples	[38]
	Logistic Regression	Easy to implement and utilise	Only two outcomes are possible	[19, 39]
	LASSO Regression	Can avoid overfitting of data	Randomly selects few highly correlated covariates with the result and shrinks the rest to 0	[40]
	Naive-Bayes	Requires a small amount of training data	Attribute independence is assumed but is often incorrect	[41]
	Random Forest	Can handle both classification and regression tasks	Computationally time demanding	[42, 43]
	k-NN	Can deal with noisy data, provided it has a large *k* value	Slow runtime	[44, 45]
Unsupervised ML	k-means Clustering	Can deal with a large amount of data	Requires advance specification on the number of data clusters	[46]
Supervised DL	CNN	Detects features without human supervision	Training requires a large amount of data	[47]
			Black box functionality (cannot understand the decision pattern behind outcomes)	
Supervised and unsupervised DL	RNN	Can be used to represent the relationship between data and time	Training is difficult	[48]
			Gradient vanishing and exploding problems	
Unsupervised DL	AutoEncoders	Reduces the dimensionality of the data used	Performance can be adversely affected if the properties of the training data is not similar to those of the testing data	[49]

SVM, Support Vector Machine; LASSO, Least Absolute Shrinkage and Selection Operator; k-NN, k Nearest Neighbour; CNN, Convolutional Neural Network; RNN, Recurrent Neural Network; ML, machine learning; DL, deep learning; AI, artificial intelligence.

The results provided by these AI algorithms are typically presented using metrics such as area under curve (AUC), area under receiver-operator characteristic (AUROC), concordance statistics (C-statistics), sensitivity, specificity or accuracy. Accuracy depicts the ability of the model to correctly identify positive cases from a dataset. Sensitivity aims to measure the amount of positives present while specificity targets the amount of negatives in the sample. AUC denotes the degree or measure of separability and is calculated by calculating the area under the curve in a graph of sensitivity [y-axis] against ‘1-specificity’ [x-axis]. An AUC of 1 stipulates that the model can categorise observations into classes perfectly while that of 0.5 performs no better than a model using random determinations [50, 51]. The C statistic calculates the likelihood that a randomly selected subject who received the result would have a higher predicted probability of receiving the result than a randomly selected subject who did not receive the result and is similar to AUROC. As in AUC, a value of less than or equal to 0.5 denotes poor performance, and a value of 1 denotes the ideal model [52, 53].

## 3. Detection and Prediction of Atrial Arrhythmias by Artificial Intelligence

### 3.1 Electrophysiology

ECG is the cheapest technique employed to confirm the presence of any atrial arrhythmia. Computerized ECG interpretation models have enhanced the physician’s ability to read ECGs more rapidly. These models are universally used in all hospitals, although they tend to be inaccurate, and over-reliance on them may lead to a wrong treatment strategy and unnecessary testing [54]. A symptomatic diagnosis of AF is confirmed by the absence of P waves and the presence of multiple fibrillatory waves between varying R-R intervals [55]. As these electrocardiographic features are sometimes absent in patients who are either at high risk of developing AF or have recurrence from a previous CA, it was crucial to develop a method which might detect changes that could indicate a high risk of recurrent AF. P wave morphology, amplitude, duration and P-R interval were identified as probable predictors of AF on ECGs and were subsequently used to detect and predict risks of developing AF [9, 10]. Even if the physician is able to use these electrophysiological features to correctly predict the likelihood of AF in high-risk patients, the ECG changes are occasionally subtle and imperceptible.

AI methodologies, on the other hand, have demonstrated the ability to identify or predict the likelihood of AF in high-risk patients by detecting very subtle changes in ECGs that may be overlooked or dismissed as a normal finding [25, 33, 56, 57, 58]. These studies paved the way for further research into the predictors of recurrent AF following CA. Tang *et al*. [59] investigated the ability of a DL model trained on a standard 12-lead ECG to predict AF recurrence one year after CA. In identifying subtle ECG changes that could be predictive of recurrent AF after CA, the model achieved an AUROC of 0.767, a sensitivity of 0.812, and a specificity of 0.770. AF drivers, which are responsible for sustaining arrhythmias, are usually targeted and terminated on CA [60]. While ECGs are usually performed after the procedure to assess their efficacy, subtle signal variations may still be present, indicating that the AF drivers have not been completely ablated. Luongo *et al*. [61] used ML techniques on a standard 12-lead ECG to distinguish between pulmonary veins (PV)-related AF drivers and non-PV drivers. Their ML classifier was able to identify subtle variations in PV driver patterns that might result in an AF recurrence after CA with a sensitivity of 73.9% and a specificity of 82.6%. Although the origin of these AF drivers can be mapped during non-invasive mapping [62], AI technology can benefit surgeons with its in-depth ability to detect very small changes that may indicate a recurrent focus for an arrhythmia, allowing surgeons to reconsider their ablation strategies.

Although the majority of AI-related studies have used standard 12-lead ECG modalities, their capabilities on continuous ECG recording devices, also known as ambulatory devices such as implantable cardiac monitor (ICM) or Holter monitoring devices, have also been evaluated [34, 63]. These devices can measure heart rate variability (HRV), including both episodes of arrhythmias and sinus rhythm, which has been identified as a useful method for predicting post-ablation arrhythmia recurrences [64]. The capabilities of AI methodologies were then investigated for predicting AF recurrences after ablation. Saiz-Vivo *et al*. [65] used ML techniques on HRV features collected from an ICM to predict AF recurrences in CA patients. The AUC of the ensemble classifier they used was 0.85, indicating that HRV features like R-R interval variability and discrepancies were predictors of post-ablation AF recurrence. The authors utilised clinical variables in addition to ECG parameters. In another study, Zvuloni *et al*. [66] used ML models on HRV features on a conventional but continuous 12-lead ECG monitor used throughout the procedure to analyse features on pre- and post-ablation ECGs to predict AF recurrence after CA. Using HRV features indicative of post-ablation AF recurrence, the model achieved an AUROC of 0.60 and 0.67 in pre- and post-ablation ECGs, respectively. The authors noted that the inclusion of demographics had raised their AUROC to 0.64 and 0.74, respectively, for pre- and post-ablation ECGs. Both studies showed that AI methodologies can detect subtle ECG changes in HRV predictive of future AF recurrences, which can be easily overlooked or dismissed as normal sinus rhythms.

AFL and AT, in addition to AF, can result in morbidity and mortality. The presence of inverted F-wave patterns in leads II, III, and a VF, as well as an upright F wave in lead V1, usually confirms the diagnosis of AFL [67]. For ATs, an ectopic P wave preceding a narrow QRS complex, with a clear baseline confirms the diagnosis [68]. ML and DL techniques have been applied to predict these atrial arrhythmias, including morphological and durational changes in P waves, and unusual changes in cycle length [69, 70], in order to identify and predict their likelihood in high-risk patients. Luongo *et al*. [27] used ML techniques to identify the locations of the three main AFL mechanisms (CTI-dependent, peri-mitral, and other LA classes) using a standard 12-lead ECG, achieving an accuracy of 76.3% and a sensitivity of 89.7%, 75%, and 64.1% in identifying the respective classes of AFL. Besler *et al*. [71] found that lead V5 displayed ECG changes that could predict the likelihood of AFL in high-risk patients with an accuracy of 98% using ML techniques. Other AI studies have focused only on classifying or detecting the different classes of arrhythmias, which include both AFL and AT, among others [72, 73, 74, 75]. All of these studies mention the possibility of AI models identifying and detecting subtle ECG changes in patients at risk of AFL or AT.

However, AI studies predicting recurrences of AFL and AT have remained elusive and uncertain. This could be due to a lack of predictors that can predispose to AFL and AT, or to their low recurrence rate when compared to AF recurrence. Wang *et al*. [76] used an ML model on 158 patients who had previously undergone CA to detect several classes of atrial arrhythmias such as AFL, AF, and AT, and identified QT dispersion and ventricular rate as important ECG features to achieve an AUC of 0.798, a sensitivity of 77.27%, and a specificity of 84.29%. They also incorporated demographic and baseline variables into their AI model in order to improve its prognostic accuracy. Given how well these atrial arrhythmias can be classified and detected, it may be possible to develop AI methodologies that can forecast their recurrences after being CA. By making the necessary adjustments, such as alterations to the ECG patterns and wave morphologies that are specific to these arrhythmias, the same principles used for AF prediction may be applied to AFL and AT.

### 3.2 Cardiac Imaging Modalities

Cardiac imaging modalities have allowed physicians to assess the extent of structural and functional changes associated with atrial arrhythmias. Echocardiographic modalities such as TTE (transthoracic echocardiography), TEE (transoesophageal echocardiography), or Doppler imaging are typically performed at the first visit [77]. TTE is used to assess cardiac anatomic structure and function, whereas TEE is typically used to evaluate blood vessels. Doppler echocardiograms measure and evaluate blood flow through the chambers and valves of the heart. Advanced modalities such as cardiac computer tomography (CCT) and CMR, are reserved for detecting more subtle changes or when clearer imaging is required. The majority of cardiac anatomic and functional changes seen on medical imaging that are associated with an increased risk of AF include increased left atrial (LA) size and volume, structural heart disease, decreased Ejection Fraction, decreased LA strain, and diastolic dysfunction [12, 78, 79]. Analysing these changes takes time and requires a thorough knowledge of both cardiac anatomy and the locations of key cardiac imaging landmarks.

Given their ability to imitate human abilities in analysing cardiac imaging modalities to identify structures of interest, regardless of which imaging modalities they were applied to, AI models, in particular DL models, have recently been introduced into clinical practice. Not only do they have the ability of integrating and processing large amounts of images, but they can also learn from intricate patterns and recognise them on these imaging methodologies much more efficiently than can humans [80, 81]. AI methodologies have shown promise in identifying relevant structural changes, especially LA size, LA volume and LA strain and atrial fibrosis, on cardiac imaging modalities that could predispose to the development of AF [32, 35, 82, 83]. Their abilities to identify structural modifications that could predict a recurrence of AF after CA have also been investigated. Miao *et al*. [84] used DL techniques on echocardiographic images to identify imaging features that could predict AF recurrence after circumferential CA. Their model demonstrated that echocardiographic features such as changes in LA volume and LAA (LA appendage) emptying velocity were predictors of AF recurrence risk with a validation AUC of 0.878. Hwang *et al*. [85] combined speckle-tracking echocardiography (STE) with DL techniques to identify imaging modality features that might be predictive of a post-CA AF recurrence. STE has the potential to quantitatively assess regional and global myocardial function, irrespective of cardiac translation and anatomic angles [86]. Their best DL model achieved an accuracy of 83.8% with a sensitivity of 85.3% and a specificity of 82.4% in classifying outcomes after AF ablation upon examining atrial strain and strain rate, both of which are measures of myocardial contractility.

Intra cardiac echocardiography (ICE) is a type of echocardiography that allows for the real-time visualisation and detection of structural changes in cardiac structures such as the PVs and interatrial septum. It is frequently used during CA because it enables proper positioning of the circular mapping catheter, which helps to guide the surgeon during the intervention, and serves as a monitoring tool for adjusting the amount of energy delivered to avoid tissue overheating, perforation, and lesion formation caused by the catheter tip [78]. This type of medical imaging has recently been the subject of AI-related studies. Akerström *et al*. [87] investigated the feasibility of an ICE-based DL model in assisting with LA mapping and ablation. With an accuracy of 69%, the model was able to correctly identify all anatomical structures such as the LA, LAA, and PVs. In a study by Schwartz *et al*. [88], ICE was also used to build an AI algorithm capable of reconstructing the anatomy of the LA for the CARTO™ system, which is an advanced imaging technology that incorporates the electroanatomic map derived from ICE, as well as real-time orientation and localisation of the catheter in the heart, in addition to computer tomography angiography (CTA). CTA is the gold standard for assessing the LA prior to CA. With Kendall’s coefficients of concordance (a non-parametric measure used for rank correlation) of 0.949, 0.926, and 0.940, respectively, the AI model was able to reconstruct the LA morphology, common ostia of PVs, and the PV antrum. Both studies were able to demonstrate that AI algorithms can aid in detecting real-time structural changes associated with the risk of recurrent AF. Further research on these modalities may help to validate the AI models’ abilities to predict changes in real-time that may be indicative of an AF recurrence after CA.

Advanced modalities such as CCT and CMR provide higher-resolution images that can isolate and visualise the entire myocardium. These details make these imaging modalities more suitable for detecting subtle changes in the atrium and PVs, that may predict the risk of future arrhythmias. Since computer tomography (CT) scans are routinely performed prior to an ablation procedure, structural and functional irregularities in the heart may alert physicians as to whether the patient is at high risk for the recurrence of arrhythmias. However, their main disadvantage is that the patients are exposed to radiation, especially if repeated tests are required [89] as well as costly. With the use of AI technology, only one test could be required, and algorithms may be able to analyse the results in order to look for structural changes that could predispose to a higher risk for the recurrence of an arrhythmia. Liu *et al*. [90] used DL techniques to develop a model to find AF triggers that are not present in the PV from CT scans to help predict the recurrence of arrhythmias following CA. When the variations in the morphologies of the LA, RA, and PVs were used as a criterion for judging which patients were at a high risk of CA recurrence, the model’s accuracy was 82.4%, sensitivity was 64.3%, specificity was 88.4%, and the AUC was 0.82. Firouznia *et al*. [26] used ML techniques to evaluate the morphologies of the LA and PVs of patients undergoing CA to determine whether these characteristics could predispose to an AF recurrence after CA. The AUC of their best model, which combined both LA and PV changes, was 0.87. Another study by Shade *et al*. [21], sought to determine whether DL models on CMR, specifically late gadolinium-enhanced CMR (LGE-CMR), could be used to predict which patients are more likely to experience recurrence after CA prior to the procedure. Using a simulated version of LGE-CMR, the DL methodology was able to extract characteristics indicative of a high risk of AF recurrence following CA, with a validation sensitivity of 82%, a specificity of 89%, and an AUC of 0.82. These three studies demonstrated that AI methodologies have been able to extract characteristics from cardiac imaging modalities to assess the risks predisposing to the recurrence of AF. However, they have all been unable to identify those characteristics that could actually predict the new onset of AF.

The future of using cardiac imaging techniques to detect or predict AFL or AT is still uncertain. This is primarily due to the fact that imaging characteristics that contribute to AF are almost identical in AFL and AT. However, in some cases, there are distinct structural and functional features that distinguish AFL and AT from AF that have been detected using these imaging methods. For example, right atrial contractile and reservoir function, flutter movements in the left posterior atrial wall, and loss of A wave on pulse wave in Doppler imaging are functional features that are characteristic of AFL [91, 92, 93]. A small study found that a small LA size can increase the risk of AT [94]. Since these studies only included on a small number of patients and the characteristics were not consistently observed in all patients experiencing these arrhythmias, more investigations are necessary before cardiac imaging can be considered to be an effective method for identifying features that may lead to the diagnosis of these atrial arrhythmias. AI modalities may be useful in this context, but they will need large datasets of images to uncover specific patterns that may be unique to these arrhythmias.

### 3.3 Risk Calculators 

Risk calculators are flowcharts that clinicians use to determine the likelihood and severity of cardiovascular diseases in patients [95]. Risk calculators in cardiac arrhythmias are used to assess the likelihood of an arrhythmia occurring and, to predict the patient demographics that predispose to recurring atrial arrhythmias. They are typically composed of a combination of cardiac imaging data, electrophysiological data, baseline demographic data such as age and gender, as well as clinical variables like hypertension, smoking status, presence of comorbidities, and the class of medications used by the patients. These risk calculators are mainly based on patient health records, known as electronic health records (EHR), because they have been digitalised and preserved in databases that are easily accessible [96]. A system of standardized data models has been developed in order to significantly decrease the number of factors to include the most important ones given the multitude of variables currently present on available records [97]. This explains why EHR indices have been streamlined by these data models, and why risk assessment tools primarily utilize the vast majority of the data contained in these electronic records. CHARGE-AF [98], FHS [15], HATCH [16], and C2HEST [17] are a few examples of validated risk assessment tools used to assess and predict the likelihood of AF in high-risk patients. The general layouts of these risk calculators are shown in Table 2 (Ref. [15, 16, 17, 98]).

**Table 2.** **An overview of validated risk calculators and their attributes used for predicting AF in high risk patients**.

Validated risk calculators	Variables used	Cohort size	Results	References
CHARGE-AF	Age, ethnicity, height, weight, BP, smoking, antihypertensive medication use, DM, HF, MI	111,475	C-statistics of 0.74 (95% CI: 0.73–0.74)	[98]
FHS	Age, BMI, sex, PR interval, HF, murmur, systolic BP, use of anti-hypertensive medication	49,599	Overall C-statistics of 0.734 (95% CI: 0.724–0.744)	[15]
HATCH	Hypertension, age, CVA/stroke, TIA, COPD, HF	692,691	AUROC of 0.771 (no 95% CI provided)	[16]
C2HEST	CAD, COPD, age, systolic HF, thyroid disease	1,047,330	AUC of 0.588 (95% CI: 0.585–591)	[17]

AF, atrial fibrillation; BP, blood pressure; DM, diabetes mellitus; HF, heart failure; MI, myocardial Infarction; BMI, body mass index; CVA, cerebrovascular accident; TIA, transient ischemic attack; COPD, chronic obstructive pulmonary disease; CAD, coronary artery disease; AUC, area under curve; AUROC, area under the receiver operating characteristic; C-statistics, concordance statistics.

These patient records contain a plethora of information, hence it was crucial only those vital demographics to integrate into these cardiac risk calculators, as shown above. The same procedure is not required for AI methods as they have the capabilities of integrating and processing a large amount of variables. Tiwari *et al*. [99] used an ML approach to investigate 200 of the most common clinical variables in EHRs to detect parameters suggestive of the occurrence of AF. Utilizing demographics, comorbidities, and other easily obtained clinical variables over a 6-month period, the ML technique yielded an AUC of 0.79 for detecting the occurrence of AF. In another study, Hill *et al*. [100] applied AI methodologies on a cohort of 2.9 million people to predict AF in a healthcare setting using data from EHRs. Utilizing the same parameters as the former study and adding time-varying factors such as blood pressure and BMI, the latter model identified high-risk patients with an AUROC of 0.827%. However, both authors acknowledged that the omission of laboratory results in their investigations may have contributed to the lower-than-anticipated predictive values of their ML models.

Laboratory variables such as NT-proBNP, C-reactive protein, and albumin have been shown to predict the development of atrial arrhythmias [101, 102, 103]. Grout *et al*. [104] found that adding a laboratory factor such as albumin to their study resulted in better performance than the above-mentioned previous studies, with their ML approach achieving a C-statistic value of 0.81 for predicting AF in high-risk patients over a period of 2 years. Bundy *et al*. [105] incorporated laboratory values, particularly cardiac biomarkers, and used ML techniques to predict a five-year risk of AF in high-risk patients. Although the CHARGE-AF risk calculator was the main focus of the study, other laboratory biomarkers such as troponin-T, NT-proBNP, serum creatinine, and ECG were also taken into consideration. The combination of the aforementioned variables nevertheless achieved a C-statistic of 0.802 in predicting a five-year risk of AF in high-risk patients, despite the fact that measurements of cardiac imaging modalities such as CMR measurements of cardiac structures, which can predispose to the development of an arrhythmia, had been omitted. Nadarajah *et al*. [23] sought to develop a risk calculator, FIND-AF, to detect and evaluate risk factors that may be involved in predisposing to the development of new AF from routinely collected data. This DL-based risk score assessor used a total of 22 predictor variables, including ECG features, laboratory variables, changes seen on cardiac imaging, and baseline clinical variables, to predict AF in high-risk patients with an AUC of 0.827. These three studies unequivocally demonstrate the importance of incorporating imaging modalities and laboratory biomarkers into AI models for predicting the likelihood of developing an arrhythmia. Incorporating these features into future AI models may improve their ability to predict demographics that predispose patients to atrial arrhythmias.

On the basis of these tested risk calculators, a different category of risk factor calculators was created specifically to predict the likelihood of AF recurrence after ablation. Among them are the risk calculators CAAP-AF [14], APPLE [106], SUCCESS [107], and ATLAS [108]. In addition to using data from EHRs similar to validated calculators, they also incorporate real-time variables. A summary of those risk calculators is shown in Table 3 (Ref. [14, 106, 107, 108]).

**Table 3.** **A summary of the risk calculators mainly involved in assessing and predicting post-ablation recurrences of AF**.

Risk calculators	Variables used	Cohort size	Results	References
CAAP-AF	Age, LA size, type of AF, CAD, number of previous antiarrhythmic drugs failed	1125	C-statistic of 0.691 (no 95% CI provided)	[14]
APPLE	Age, type of AF, impaired eGFR, LA size, systolic LVEF	1406	AUC of 0.634 (95 % CI 0.600–0.668)	[106]
SUCCESS	Type of AF, no. of unsuccessful CA, impaired eGFR, LA size, systolic LVEF	192	AUC of 0.657 (no 95% CI provided)	[107]
ATLAS	Age, sex, type of AF, smoking, LAV	1934	C-statistics of 0.75 (no 95% CI provided)	[108]

AF, atrial fibrillation; LA, left atrium; CAD, coronary artery disease; CA, catheter ablation; LVEF, left ventricular ejection fraction; eGFR, estimated glomerular filtration rate; LAV, left atrial volume; C-statistics, concordance statistics.

Numerous clinical factors have been identified as predictors of AF recurrence after ablation. To lessen physicians’ workload, a method for identifying the most significant predictors was necessary. This resulted in the creation of the risk calculators shown in Table 3. However, as the number of variables is reduced, the possibility of predicting these events decreases. With AI technology, tailoring these clinical variables is no longer necessary because these algorithms can process, analyse and interpret a much larger amount of data to predict a possible recurrence. Hung *et al*. [109] used ML techniques to predict AF recurrence 30 days after CA. The ML model achieved an AUC of 0.91 by predicting AF recurrence using simple clinical variables such as age, gender, length of stay, hospital discharge procedures, presence of chronic diseases, and a number of diagnoses. The authors did not use ECG, medical imaging, or laboratory parameters, and they did not consider predicting AF recurrences beyond one month. Therefore, more clinical variables are needed to estimate post-ablation arrhythmia recurrences following a longer time period.

In order to predict AF recurrence after CA over a longer time period, more variables predictive of AF recurrence after CA were included. Most AI models began to incorporate electrocardiographic features, findings from medical imaging techniques, and laboratory variables, in addition to easily acquired clinical variables (baseline demographic data such as age and gender, as well as clinical variables like hypertension, smoking status, the presence of comorbidities, and the class of medications used by the patients), to achieve a better result. The presence of the aforementioned clinical variables in the post-ablation risk calculators, as shown in Table 3, supports this assertion. Researchers may utilize these risk calculators as a reference to improve the prognostic capabilities of their own AI systems by incorporating more clinical parameters. For instance, Lee *et al*. [24] assessed clinical variables made up of clinical predictors obtained from these risk calculators using ML methodologies to pinpoint the characteristics that indicate late recurrence after radiofrequency catheter ablation (RFCA) in patients with AF. The addition of the left ventricular mass index to their algorithm improved the prognostic value of their algorithm, yielding an accuracy of 0.768 and an AUROC of 0.766. In another study, Zhou *et al*. [110] used a DL-based approach to predict one-year AF recurrence after CA by adding other variables such as NT-proBNP, left ventricular mass index, and left atrial appendage volume (LAAV) to the already validated predictors of AF recurrence located on these risk calculators. This improved the C-index (C-statistics) of the DL algorithm to 0.76. It can be inferred from the results of all the studies that increasing the amount of laboratory, electrophysiological, and clinical imaging data may increase the precision with which risk calculators can identify characteristics that predispose to post-ablation recurrent AF over a longer period of time.

As a result of these findings, risk calculators that use AI technology to predict features that predispose to post-ablation arrhythmia recurrences were developed. For example, the previously mentioned FIND-AF [23] risk calculator can be customized to predict post-ablation recurrence of AF on a given timeline because it contains all the components required to predict AF in high-risk patients. Other ML-based risk calculators have been developed. Saglietto *et al*. [111] created AFA-RECUR, an ML-based risk score calculator, by combining 19 variables, including baseline and simple clinical variables, laboratory, electrophysiological, and clinical imaging data. In predicting a 1-year AF recurrence after CA, the model had an AUC of 0.721. Furthermore, they have implemented a system that quantifies the likelihood of recurrence based on data input by patients into the model, classifying it as low or high. STAAR, another ML-powered risk score calculator developed by Park *et al*. [112] produced AUCs of 0.935, 0.855, and 0.965 for categorizing high-risk patients as having a low, medium, or high probability of AF progression to permanent AF after CA. The assignment for the score criteria was determined by the severity of the factors that are suggestive of AF. It is evident from these studies that combining clinical factors increases the accuracy of predicting post-ablation recurrences of AF, and how this will enhance AI models and create more features suggestive of post-ablation recurrences, allowing the development of new risk calculators.

In comparison to AF, risk calculators involved in diagnosing and forecasting AFL and AT are more challenging. The determinants involved in identifying or predicting the likelihood of AFL are comparable to those of AF because of the common risk factors they share. Aside from the aforementioned electrophysiological changes, patients with structural heart disease or respiratory disease are more likely to develop AFL [113]. A few distinguishing features that could separate these arrhythmias after CA have been observed: amiodarone use prior to CA, deeper lesions following the ablation procedure, and a fluoroscopy time of more than 50 minutes were found to promote post-ablation AFL recurrence [114, 115], while cardiac imaging revealed a thicker isthmus myocardium, and abnormalities in the right atrium and right coronary artery [91, 114, 116] post ablation. In AT, a study found that patients with a smaller LA volume and no hypertension were likely to have recurrent arrhythmias following CA [94]. However, these features have not been unanimously reported in patients experiencing these arrhythmias and have been observed only in small cohorts. More precise clinical characteristics are needed to properly distinguish AFL and AT from AF.

The scarcity of AI studies detecting and predicting the likelihood AFL and AT from clinical data reflects the limited number of factors distinguishing them. However, because of the vast amount of clinical data available on EHRs, researchers have opted for AI methodologies to detect features that might predispose to the development of AFL or AT. Kim *et al*. [117] found that combining patient history data, ECG, and clinical imaging features to predict features that could predispose to the development of AF and AFL from asymptomatic atrial tachyarrhythmia detected by cardiac implantable electronic devices with atrial sensing (AHRE) resulted in an AUROC of 0.745. In a different study by Hill *et al*. [118] neural networks were used to find characteristics of high-risk patients in a cohort of 3 million patients that were suggestive of AF and AFL. Their ML technique produced an AUC of 0.907 using clinical data from EHRs, as well as time-varying covariates like blood pressure. However, both studies included AF rather than an isolated study of AFL and AT. Hence, more independent studies are needed.

Further investigations will be critical to determine distinct clinical features that distinguish these arrhythmias from AF before a risk calculator capable of identifying or predicting the likelihood of AFL and AT is developed. AI models have the capacity to process sizable amounts of clinical data from EHRs, and as a result, they can assist in identifying traits that are more pronounced in those arrhythmias. Therefore, ML and DL methodologies may be of great assistance in this field.

## 4. Limitations of AI Studies

Although ML and DL models have successfully detected and predicted atrial arrhythmias using baseline and easily acquired clinical variables, electrophysiological features, laboratory measures, and measurements obtained from cardiac imaging, they still have several limitations that require further investigation. The most significant limitation, regardless of modality, was that many studies involved small patient cohorts. The main reason for the drastic decrease in sample size is the exclusion of “imperfect data”, which is raw data that does not appeal to researchers. As a result, these models are unable to be validated in clinical settings. To validate ML and DL models in a clinical setting, larger-scale studies with a larger cohort, as well as the inclusion of more clinical variables, should be considered.

There are restrictions that are exclusively associated with the type of modalities in which the AI studies were performed. Although studies have made use of raw ECG signals, they tended to withdraw those with noise and artifacts. The removal of ECG signals with noise and artifacts was done to allow for smooth signal analysis when AI models are used. However, ECG signals in a real-world setting are accompanied by noise and artifacts which presents a significant limitation for electrophysiological studies when AI models are used. As a result, developing dynamic AI models that can consider such signals in order to mimic real-life situations will be pivotal. Another limitation in these studies is the generation of false-positive results [119]. To counter this issue, a large number of ECG patterns can be used to train the models, increasing their exposure to patterns of interest. Continuous updates will increase their reliability and act as a self-audit. This will enhance the ability to perform statistical analysis on previous data in which results were incorrectly reported.

In AI studies using cardiac imaging, the models had to deal with image classification issues such the high dimensionality of data (huge quantity of features present on images) and the lack of labelled data [120]. The models are exposed to underfitting, in which insufficient data was included in the algorithms to enable them to distinguish between normal and diagnostic images, or overfitting, where too many characteristics were fed into the models but were not discovered during image analysis, leading to incorrect outcomes [121]. It may be difficult to overcome this problem due to the subjective nature of imaging unless standardised imaging techniques and modalities can be maintained. The lack of published detailed methods and quantitative results further restricts researchers from comprehending issues related to overfitting or underfitting. However, according to Feldner-Busztin *et al*. [120], the problem of high dimensionality may be resolved by decreasing, choosing, and extracting (compacting features into a user-specified number of new features) the number of relevant features from the total number gathered.

It must be acknowledged that recurrence following ablation therapy is highly dependent on the surgeon’s skill [122]. In cryoablation for AF, there is little variation in treatment results between surgeons, unless it is during the learning curve [123]. In contrast, radiofrequency (RF) ablation success rates vary depending on the surgeon’s skill. While AI algorithms have been shown to detect minute signal changes and subtle changes on cardiac imaging that are indicative of atrial arrhythmias, they cannot determine the surgeon’s skills. They can only recommend which ablation strategy might be more effective based on the type of atrial arrhythmia or the patient’s condition. A virtual reality (VR)-based surgical skills training simulator with real-time settings has recently been developed to assist surgeons in learning these techniques [124]. As a result of VR, AI can only interact with the surgeon to aid during CA but, it cannot interfere with the skills of the surgeon.

One would argue that AF is the most pertinent atrial arrhythmia among all, and there is a lack of discerning features to distinguish between them, explaining why AI studies on AFL and AT were limited. Nonetheless, the authors of this manuscript have attempted to summarize several discerning features such as changes in ECG, characteristics measured during cardiac imaging like loss of A-wave on pulse-wave in Doppler imaging and contractility of the right atrium, and a smaller LA (that is significant of AT) that could differentiate AFL and AT from AF [7, 67, 68, 93]. The authors have outlined a few studies that used clinical variables and electrophysiological features to identify characteristics that might be predisposing to AFL and AT [27, 72, 73, 74, 75, 76], and they have conjectured about how specific features seen or measured on cardiac imaging might be useful in artificial intelligence studies to identify features present in high risk patients who might develop such arrhythmias [91, 92, 93, 94]. This has led to the conclusion that electrophysiological features are critical for differentiating the arrhythmias, pointing out that most AI-related studies could explore this field to predict post-ablation recurrence of these arrhythmias. Studies involved in AFL and AT have collided with the same limitations as those found in AF, implying that further research will be necessary in order to achieve better results.

## 5. Conclusions

As AI technology advances and our knowledge of its uses expands, we can ascertain conclusively that AI will become more significant in medicine and will unquestionably become a great tool for doctors. Although these AI models have limitations and are still in their early stages, their capacity to analyse and interpret a large amount of data to forecast atrial arrhythmias demonstrates great promise. This suggests that additional investigation into developing more effective strategies to get around these challenges is required before their eventual deployment in a clinical environment. Additionally, it was mentioned that the combination of several traits can help forecast recurrences over a longer period of time and more accurately. The authors believe that the ability of these AI algorithms to detect and predict AF, AFL, and AT will definitely be improved by the integration of clinical variables, relevant imaging findings, and electrophysiological data in the future.
